# NADPH Oxidases Connecting Fatty Liver Disease, Insulin Resistance and Type 2 Diabetes: Current Knowledge and Therapeutic Outlook

**DOI:** 10.3390/antiox11061131

**Published:** 2022-06-09

**Authors:** Alberto Nascè, Karim Gariani, François R. Jornayvaz, Ildiko Szanto

**Affiliations:** 1 Service of Endocrinology, Diabetes, Nutrition and Patient Therapeutic Education, Geneva University Hospitals, Rue Gabrielle-Perret-Gentil 4, 1205 Geneva, Switzerland; alberto.nasce@hcuge.ch (A.N.); karim.gariani@hcuge.ch (K.G.); 2 Department of Medicine, Faculty of Medicine, University of Geneva, 1211 Geneva, Switzerland; 3 Diabetes Center of the Faculty of Medicine, University of Geneva Medical School, 1211 Geneva, Switzerland; 4 Department of Cell Physiology and Metabolism, Faculty of Medicine, University of Geneva, 1211 Geneva, Switzerland

**Keywords:** hepatosteatosis, nonalcoholic fatty liver disease, NAFLD, reactive oxygen species, ROS, NADPH oxidase, NOX, insulin resistance, diabetes, oxidative stress

## Abstract

Nonalcoholic fatty liver disease (NAFLD), characterized by ectopic fat accumulation in hepatocytes, is closely linked to insulin resistance and is the most frequent complication of type 2 diabetes mellitus (T2DM). One of the features connecting NAFLD, insulin resistance and T2DM is cellular oxidative stress. Oxidative stress refers to a redox imbalance due to an inequity between the capacity of production and the elimination of reactive oxygen species (ROS). One of the major cellular ROS sources is NADPH oxidase enzymes (NOX-es). In physiological conditions, NOX-es produce ROS purposefully in a timely and spatially regulated manner and are crucial regulators of various cellular events linked to metabolism, receptor signal transmission, proliferation and apoptosis. In contrast, dysregulated NOX-derived ROS production is related to the onset of diverse pathologies. This review provides a synopsis of current knowledge concerning NOX enzymes as connective elements between NAFLD, insulin resistance and T2DM and weighs their potential relevance as pharmacological targets to alleviate fatty liver disease.

## 1. Introduction

Nonalcoholic fatty liver disease (NAFLD) is a chronic liver disease that encompasses a spectrum of diverse pathologies, ranging from simple steatosis to nonalcoholic steatohepatitis (NASH), fibrosis and cirrhosis, and eventually hepatocellular carcinoma (HCC) [1]. It is increasingly recognized that NAFLD is associated with insulin resistance and type 2 diabetes (T2DM) [2]. Indeed, in insulin-resistant states, excessive circulating free fatty acids (FFAs) are delivered and sequestered in ectopic fat depots, with preferential accumulation in hepatocytes. FFAs promote endoplasmic reticulum (ER) stress, inflammation and the production of reactive oxygen species (ROS) leading to the onset of oxidative stress [3,4]. These cellular events, in turn, further aggravate insulin resistance, perpetuating a vicious cycle. The pathological implication of redox imbalance in the development of NAFLD, insulin resistance and T2DM is denoted by many preclinical studies [5,6]. However, despite extensive investigations, the therapeutic application of generic antioxidant molecules in human trials has not led to convincing results [4]. The failure of this generalized, “all-out” approach might lie in the complexity of the interactions and compensatory mechanisms that govern cellular redox homeostasis [7,8,9]. Accordingly, the current understanding promotes a more nuanced attitude by targeting individual pro- and antioxidant elements and thus lessening the specific pathological processes that are most relevant for each disease [10]. In this respect, nicotinamide adenine dinucleotide phosphate (NADPH) oxidase enzymes, NOX-es, have gained significant interest due to their wide-scope implications in physiological signaling pathways and their regulated ROS-producing activity [11,12]. Indeed, NOX enzymes produce ROS as their only enzymatic activity, as opposed to other cellular ROS sources that generate ROS as secondary byproducts along with their main enzymatic function [13]. However, when uncontrolled, NOX-derived ROS generation is associated with the onset of diverse metabolic pathologies, in particular NAFLD, insulin resistance and T2DM [4]. Each stage of NAFLD, stretching from steatosis to HCC, has been shown to be related to disturbances in diverse NOX enzyme-mediated ROS production [4,13]. A recent excellent review provided a detailed overview specifically focusing on the role of NOX enzymes in TGF-β signaling, which is a key factor in the development of these later stages of hepatic pathologies [14]. The present review concentrates on the implications of NOX enzymes as links between hepatic steatosis, insulin resistance and T2DM, and summarizes the current knowledge about their relevance as potential therapeutic targets in NAFLD.

## 2. Liver Redox Homeostasis and NOX Enzymes

### 2.1. Liver Redox Homeostasis

Redox homeostasis refers to an equilibrium between the production of ROS and their degradation or neutralization by components of the antioxidant network. ROS are short-lived but chemically highly reactive radicals or molecules that are produced in a continuous fashion by diverse enzymes as byproducts of their physiological function [15]. ROS, however, can also be produced in a purposeful manner by a specific class of enzymes, termed NADPH oxidases (NOX-es) and dual oxidases (DUOX-es) [16]. The major cellular ROS types are superoxide (O_2_^•−^), hydrogen peroxide (H_2_O_2_) and hydroxyl radical (OH^•^) [17]. In hepatocytes, superoxide production is attributed to mitochondria and to specific NOX enzyme isoforms (NOX1, NOX2, NOX3 and NOX5). In the mitochondria, electron leakage in the complex I and III of the electron transport chain (ETC) can lead to superoxide formation and this phenomenon is specifically relevant during periods of substrate overload. In addition, other mitochondrial metabolic enzymes (α-ketoglutarate dehydrogenase, pyruvate dehydrogenase, glycerol phosphate dehydrogenase, and monoamine oxidase) produce superoxide linked to their physiological activity [18]. Superoxide is also generated by diverse non-mitochondrial enzymes, namely xanthine oxidase, cytochrome P450 2E1, cyclooxygenases and lipoxygenases [8]. Hydrogen peroxide is derived mainly from peroxisomal fatty acid oxidation, but it is also produced directly by the NOX isoform NOX4 and two DUOX enzymes (DUOX1 and DUOX2) [15,19]. In addition, H_2_O_2_ is generated as an intermediary molecule during superoxide detoxification [20]. Hydroxyl radicals are produced by a ferric ion (Fe^2+^)-catalyzed Fenton reaction with H_2_O_2_ [21]. Superoxide and H_2_O_2_ are considered signaling entities with specific molecular targets and lower chemical reactivity while (OH^•^) is related to pathological processes due to its high level and non-discriminative oxidative capacity [22,23,24].

Cellular ROS are rapidly eliminated by diverse antioxidant enzymes/systems to prevent redox injury to biomolecules and intracellular organelles [9,20]. In addition, the timely removal of physiological signaling of ROS is crucial for terminating receptor activation events [25]. The elimination of ROS is mediated by several intertwined antioxidant pathways. The components of this network are superoxide dismutase (SOD), catalase and the glutathione (GSH and thioredoxin (Trx)/peroxiredoxin (Prx) systems. The different SOD enzyme isoforms (SOD1 in the cytosol, SOD2 in the mitochondria and SOD3 in the extracellular space) convert superoxide anions to H_2_O_2_ and molecular oxygen [26,27,28]. The relevance of SOD enzymes in liver function and in NAFLD in particular, is supported by several preclinical studies. For example, treatment of high-fat diet (HFD)-fed mice with nanoformulated SOD decreased plasma triglyceride (TG) levels and lessened hepatic steatosis. The livers of these mice displayed attenuated mRNA levels of fatty acid synthase (FAS), a key enzyme of de novo fatty acid (FA) synthesis [29]. In another study, Cui et al. demonstrated that HFD-fed mice transduced with adenoviral SOD vectors displayed diminished liver accumulation of TGs and cholesterol, along with attenuated expressions of several de novo lipogenesis (DNL) genes (Sterol regulatory element-binding transcription factor 1 (*SREBP1c*), stearoyl-CoA desaturase 1 (*Scd1*), and Fatty Acid Synthase (*Fasn*)). In addition, mice with SOD overexpression presented a lessened adipose tissue pro-inflammatory gene expression signature (Tumor Necrosis Factor α (*Tnfa*), Monocyte Chemoattractant Protein 1 (*Mcp1*) and Interleukin 6 (*Il6*)) [30]. De novo lipogenesis and inflammation are two of the major elements involved in the development of NAFLD in humans underlying the potential relevance of disturbed SOD function the accompanying metabolic disturbances [31,32].

The second step of superoxide detoxification is carried out by catalase, which reduces H_2_O_2_ to water and molecular oxygen [33]. Catalase is responsible for the elimination of about 50% of the cellular H_2_O_2_ produced [33]. Catalase is an integral component of the peroxisomal and mitochondrial antioxidant defensive systems, assisting in the maintenance of physiological steady-state H_2_O_2_ levels [34,35]. Peroxisomes and mitochondria are critical organelles in FA metabolism as short-, medium- and long-chain FAs are degraded in the mitochondria (β-oxidation), while the toxic, very long-chain FAs are oxidized within peroxisomes. Peroxisomes and mitochondria interact, and their redox status can reciprocally modify each other’s function [36]. The metabolic consequences of inadequate catalase function were demonstrated in mice with catalase deficiency (Catalase knock-out (CKO) mice). Indeed, the CKO mice presented enhanced liver TG accumulation and elevated expressions of collagen and inflammatory cytokines accompanied by the onset of oxidative stress. The underlying cause was identified as insufficient hepatic peroxisome biogenesis that triggered the development of ER stress and the upregulation of genes related to DNL. In addition, livers of CKO mice showed attenuated insulin-receptor signaling, demonstrating the critical role of catalase as a connecting element between redox homeostasis, liver lipid handling and insulin resistance *in vivo* [37]. Concerning humans, Goth et al. reported that subjects with hypo- or acatalasemia present a predisposition toward the development of diabetes mellitus [38].

Other H_2_O_2_-eliminating pathways include glutathione (GSH) and Trx/Prx systems. These systems operate in a coordinated and cyclic manner, utilizing subsequent oxidation/reduction reactions to detoxify cellular H_2_O_2_ [39,40,41]. Glutathione is a tripeptide (γ-glutamyl-cysteinyl-glycine) and is the most abundant soluble antioxidant in the cell that exists in thiol-reduced (GSH) and disulfide-oxidized (GSSG) forms [42]. GSH is indispensable for the maintenance of the cellular redox balance and metabolic function, and it plays a role in the modulation of apoptosis/necrosis. The liver has the highest GSH concentrations (approximately 10–15 mM) among all organs, reflecting the importance of GSH in diverse hepatic antioxidant, metabolic and detoxification processes [43]. GSH is synthesized in hepatocytes that provide the necessary GSH supply for other organs through transport in the bile or in the sinusoidal blood. Therefore, hepatic GSH synthesis is one of the determining factors for whole-body redox balance [44,45]. GSH is synthetized in the cytosol, but subsequently it is also transported into the mitochondria, the nucleus and the ER with a final distribution of about 85% of GSH in the cytosol and 10–15% in the mitochondria [45,46]. The enzymes participating in GSH synthesis are the glutamate-cysteine ligase (GCL) and GSH synthetase (GS), whose functions are modulated by various pathways, among them transcriptional upregulation through redox-regulated transcription factors, e.g., the nuclear factor-(erythroid factor 2)-related factor 2 (Nrf2) and nuclear factor kappa-light-chain-enhancer of activated B cells (NFκB) [43]. Reciprocally, nuclear GSH is crucial for the maintenance of the nuclear reductive milieu to allow the proper activation of these transcription factors [47]. GCL is composed of two subunits, a larger catalytic (GCLC) and a smaller modifier (GCLM) subunit, which are encoded by two separate genes [48,49]. GLCL is catalytically active on its own, but its association with CLCM enhances its activity by lowering the Km for glutamate and increasing the Ki for GSH [50,51]. In the GSH/GSSG system, the initiation step of H_2_O_2_ detoxification is the reduction of H_2_O_2_ by glutathione peroxidase (GPX), accompanied by concurrent oxidation of the free sulfhydryl (-SH) groups of glutathione (GSH), converting it into glutathione disulfide (GSSG). In the next step, the oxidized glutathione (GSSG) is again reduced by glutathione reductase (GR), utilizing NADPH as a reducing agent. The resulting GSH can then be employed in the next H_2_O_2_ detoxification cycle. Under physiological conditions, the GSH/GSSG ratio is maintained at approximately 100:1, but under oxidative duress, it can decrease to levels as low as 10:1 [43,45].

Altered functions of the enzymes of the GHS synthetic pathways are connected to disturbed hepatic lipid metabolism and the development of steatosis and insulin resistance in a multifaceted manner. Indeed, whole-body homozygous deletion of the *gclc* gene coding for the catalytic domain of GCL in mice results in embryonic lethality, while heterozygous mice are viable but display increased levels of the antioxidant ascorbate, likely to compensate for lower GSH amounts [52,53]. The central role of GSH in hepatocyte redox and metabolic balance is supported by data obtained from mice with hepatocyte-specific deletions of *gclc* [54]. Indeed, mice with homozygous hepatocyte-specific deletion of *gclc* develop fatty liver due to mitochondrial injury, and die around the age of 1-month after developing liver failure. On the contrary, mice with knock-out for *glcm* coding for the modifier subunit of GCL are protected against the development of HFD- or methionine-choline deficient diet (MCD)-induced steatosis, inflammation and fibrosis, and they maintain insulin sensitivity [55,56]. This protective effect was attributed to metabolic adaptation with attenuated expressions of lipogenesis-promoting genes (*Scd1/2*, *Fasn*, peroxisome proliferator activated receptor-α (*Ppara*) and peroxisome proliferator-activated receptor γ coactivator-1 (*Pprgc1*), along with enhanced activity of antioxidant enzymes (GPX and GSR). The critical role of GSH in early development was further substantiated by a study by Winkler et al. that demonstrated the embryonic lethality of homozygous deletion of the GS-coding *gss* gene [57]. Interestingly, however, mice with heterozygous mutation (*gss*^+/−^ mice) displayed no apparent phenotype under non-stressed conditions. In these mice, the protein amount and activity of GS was approximately 50% of that of control, wild type (WT, *gss*^+/+^) mice but the amount of liver GSH was unaltered. Taken together, these preclinical studies provide evidence that complete abrogation of GSH synthesis is incompatible with embryogenesis; however, heterozygous deletion of GSH synthetic enzymes can be compensated through different metabolic pathways. In humans, deficiency in GSH synthetic enzymes is extremely rare and the associated pathologies are incompletely understood. For example, in a cohort of 401 patients with T1DM, the 129C/T single nucleotide polymorphism, resulting in lower promoter activity for the catalytic subunit of GCL, was related to more severe renal pathology [58]. GS deficiency is a rare autosomal recessive genetic disease that leads to childhood death in about 25% of patients due to various pathologies, including acidosis, electrolyte imbalance, and convulsions [59]. Direct, specific links between altered functions in GSH synthetic enzymes and NAFLD have not been identified in humans.

The enzymes participating in the GSH/GSSG detoxification cycle are GPX and GR. There are 8 GPX isoforms that incorporate either selenocystein or cysteine in their redox catalytic centers. GPX are present in diverse organs and intracellular compartments, including the cytosol, mitochondria and the nucleus [60]. The isoform GPX1 is the dominant isoform expressed in the liver. Mice with whole-body deletion of the *Gpx1* gene are protected from HFD-induced steatosis and liver damage attributed to improved hepatic insulin sensitivity [61]. The direct role of GPX1 in hepatocytes was addressed in another study employing a mouse model with hepatocyte-specific deletion of GPX1 [62]. Hepatocytes isolated from GPX1-deficient mice produced increased quantities of H_2_O_2_ and presented a decreased GSH/GSSG ratio. Unexpectedly, however, GPX1-deficient mice were protected from HFD-induced liver insulin resistance and inflammation and maintained hepatic insulin sensitivity. In addition, the administration of MCD resulted in mitigated liver fibrosis in GPX1-deficient mice when compared to WT mice. Taken together, these data imply that hepatocyte H_2_O_2_ is in fact an essential component of liver insulin signaling and exerts a protective effect against diet-induced NAFLD/NASH. Therefore, upregulation of H_2_O_2_ observed in hepatic pathologies might actually represent a compensatory effect attempting to maintain proper liver functioning [62]. The essential and complex role of another GPX isoform, GPX4, in liver function was demonstrated by Carlson et al. [63]. Indeed, homozygous deletion of *Gpx4* in mice results in embryonic lethality [64]. By contrast, mice with hepatocyte-specific GPX4 deletion are viable but present extensive hepatocyte damage at birth and die shortly afterwards [63]. The complexity of the maintenance of cellular redox balance is indicated by data revealing that the embryonic lethality of *Gpx4* abrogation could be prevented by vitamin E administration to pregnant females and that vitamin E supplementation to mice with hepatocyte-specific *Gpx4* deletion could prevent neonatal death [63]. To investigate the role of GR, mice with GR deletions were created. GR-deficient mice presented with diverse lung abnormalities, but their liver function was not assessed [65]. The implication of the GSH/GSSG redox system in liver pathologies is multifaceted and sparked interest as a potential therapeutic target in NAFLD; however, the clinical relevance of such approaches is currently not established [66,67,68].

Next to catalase and the GSH/GSSG reducing couple, the other major H_2_O_2_ elimination system is the peroxiredoxin/thioredoxin system. Peroxiredoxins are small thiol-containing proteins that reduce H_2_O_2_ to H_2_O while themselves undergo oxidation at their cysteine residue(s) within their catalytic centers [69,70]. Oxidized Prx proteins are subsequently reduced by thioredoxins (Trx), a family of small disulfide-containing proteins. Thioredoxins are regenerated by the thioredoxin reductase (TrxR) enzyme in an NADPH-dependent reaction [71]. Six mammalian Prxs, termed Prx1–6, are localized in different intracellular compartments [72]. Prx1 and 2 are predominant in the cytosol and nucleus [73]. Prx3 is localized in the mitochondria, Prx4 in the ER, Prx5 in the mitochondria, the peroxisomes and the cytosol, while Prx6 is present in the cytosol and lysosomal compartments [72,73,74]. The role of Prxs in the defense mechanism against liver metabolic redox damage has been supported by several studies. For example, Prx5 overexpression alleviates FFA-induced lipid accumulation in HepG2 cells in vitro and in the livers of HFD-fed mice *in vivo* by mitigating the expression of the lipogenic transcription factor SREBP-1 and lessening FFA-induced mitochondrial ROS generation [75]. Similarly, Prx4 and 6 protect against steatosis by alleviating ROS production in the mitochondria [76,77]. In addition, Prx6-deficient mice display dyslipidemia with elevated TG and very low-density lipoprotein (VLDL) levels along with increased expressions of pro-inflammatory cytokines in white adipose tissue (WAT), demonstrating the complex role that Prx proteins play in the connections between hepatosteatosis, metabolic disorders and inflammation [78,79]. Taken together, the constituents of the diverse cellular antioxidant systems form a complex, intertwined network and the integrity of this network is crucial for the maintenance of healthy cell function [80]. Indeed, in serum samples of patients with NAFLD or NASH, the activity of several antioxidant enzymes (SOD1, GPX, GR) were elevated and signs of cellular oxidative damage were evidenced (increased malonylaldehyde (MDA) levels and DNA/RNA damage) [81].

The NADPH employed by the above detailed antioxidant systems is derived from diverse sources, the most significant ones being the pentose phosphate, the α-ketoglutarate/isocitrate and the folate-mediated one-carbon pathways catalized by the glucose-6-phosphate dehydrogenase (G6PDH), the isocitrate dehydrogenase (ICDH) and the methylenetetrahydrofolate dehydrogenase (MTHFD) enzymes, respectively. The cellular NADP^+^/NADPH ratio is a critical component of both hepatocyte redox balance and lipogenesis that is regulated through a multitude of interacting signaling pathways [82]. The detailed description of these metabolic pathways exceeds the scope of this review, but has been excellently summarized in several recent publications [66,82,83].

**Figure 1:**
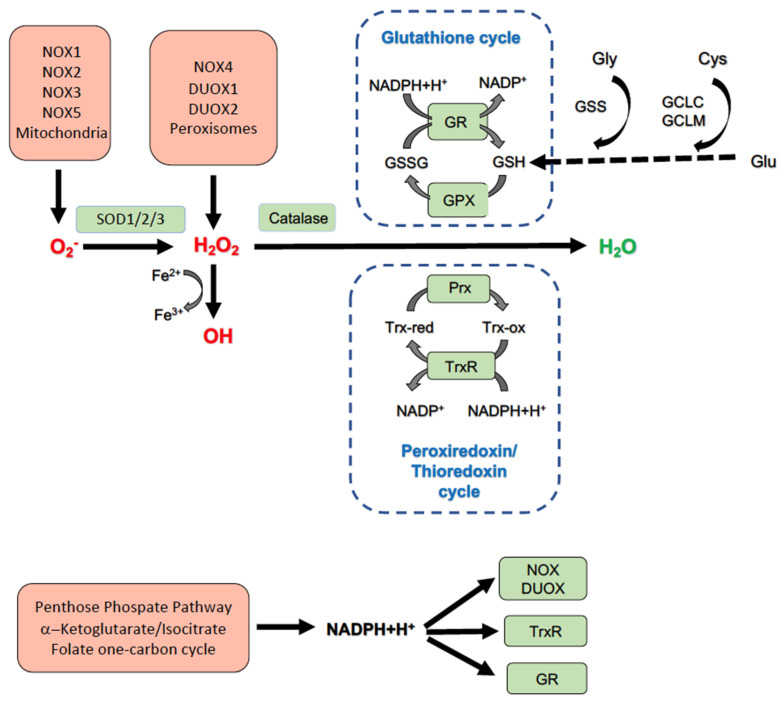
Cellular redox homeostasis. The main sources of cellular superoxide (O_2_^−^) are mitochondria and NADPH oxidase (NOX) enzymes. Hydrogen peroxide (H_2_O_2_) is produced by the peroxisome NOX4 and the two dual oxidase (DUOX) enzymes. O_2_^−^ is transformed by superoxide dismutase (SOD) into H_2_O_2._ H_2_O_2_ elimination can occur through different enzyme systems that allow for the conversion of H_2_O_2_ into H_2_O. In addition, H_2_O_2_ can give rise to a highly toxic hydroxyl radical (OH) in a metal-catalyzed reaction. NADPH is replenished from different cellular metabolic sources. GPX: glutathione peroxidase; GSH: Reduced form of glutathione; GSSG: glutathione disulfide, oxidized form of glutathione; GR: glutathione reductase; NADPH: Nicotinamide adenine dinucleotide phosphate; Prx: Peroxiredoxin proteins; Trx-red: Reduced form of thioredoxin; Trx-ox: Oxidized form of thioredoxin; TrxR: thioredoxin reductase.

The elements substantiating the cellular redox balance are shown in Figure 1.

Preservation of redox homeostasis is primordial to healthy hepatocyte functions [84]. Perturbations in the liver redox balance will lead to the exhaustion of compensatory redox buffer capacity, triggering the onset of oxidative or reductive stress [85,86]. Both of these stress conditions exert adverse effects on hepatocytes and are linked to the onset of impaired metabolic processes characteristic of NAFLD [4,87,88]. Indeed, cellular oxidative stress due to excess FA-derived substrate overload in the mitochondrial ETC or in the peroxisomal β-oxidation system will lead to further mitochondrial and peroxisomal damage triggering a vicious cycle between excessive metabolic efflux, pathological ROS production and organelle dysfunction [89,90]. On the contrary, reductive stress, referring to the accumulation of reducing equivalents (NADH, NADPH and GSH), will decrease cellular ROS levels below their physiological quantities, thus perturbing ROS-mediated signaling events (e.g., receptor signaling, transcription factor activation) [88]. For example, in rat primary hepatocytes, hypoxia led to an increase in the cytosolic NADH/NAD^+^ ratio (indicating the onset of reductive stress), which correlated with hepatocyte injury, independent of ATP depletion [91]. In addition, reductive stress triggers ER stress and activates the Unfolded Protein Response (UPR). Indeed, the ER is an oxidative environment that is necessary for proper disulfide bond formation and folding of membrane and transport proteins [92]. Under reductive conditions, proteins are inappropriately folded and accumulate in the cell, which can lead to cellular stress and the activation of apoptotic pathways [93].

Studies have demonstrated that several components of this complex and fine-tuned redox regulatory network are connected to the onset of both insulin resistance and NAFLD. For example, exhaustion of SOD2 and GPX antioxidant abilities in mice aggravated insulin resistance and the progression of NAFLD to steatohepatitis and fibrosis [94,95,96]. By contrast, lowering hepatocyte cytosolic NADH levels *in vivo* in HFD-fed mice through genetic manipulations resulted in improved hepatic insulin resistance and amended metabolic parameters independent of the classical insulin receptor signaling pathway [97]. In addition, elevated hepatocyte ROS levels result in ER stress and cell death that can provoke the activation of the key fibrogenic liver cells, the hepatic stellate cells (HSC), as well as the resident macrophages termed Kupffer cells (KCs) by various mechanisms, including apoptotic bodies and damage-associated molecular patterns (DAMPs). Activation of HSCs results in excessive extracellular matrix production, setting the scene for the development of liver fibrosis, which could then progress further to cirrhosis and cancer [98,99,100]. In NAFLD and NASH patients, the constituents of redox homeostasis are often altered [4,81].

### 2.2. NOX Enzymes in the Liver

NOX/DUOX family enzymes (reviewed extensively in [13]) are six-transmembrane proteins that are homologues of the phagocyte NADPH oxidase, originally termed gp91^phox^, and later renamed NOX2. The general structure of NOX-es is comprised of a conserved core element with four heme-binding histidines, allowing electron transport across membranes and two cytoplasmic C-terminal sites that bind NADPH and flavin adenine dinucleotide FAD. There are five NOX and two DUOX isoforms known as NOX1, NOX2, NOX3, NOX4 and NOX5, and DUOX1 and DUOX2, respectively. NOX1, 2, 3 and 5 generate superoxide, while NOX4 and the two DUOX enzymes produce H_2_O_2_. NOX1, 2 and 3 require additional cytosolic organizer and activator factors for functioning, NOX5 and the two DUOX enzymes are activated by Ca^2+^, while NOX4 produces ROS in a continuous manner. All NOX isoforms are found in most mammals, including humans, apart from NOX5, which is absent from the mouse and rat transcriptomes. NOX-es are present in all organs, with an exclusive expression pattern defined by cell type- and intracellular organelle-specific localizations. In addition, the ROS-producing activity of NOX-es is strictly regulated by distinctive extra- and intracellular signals in a timely and spatially controlled manner. This regulated ROS production distinguishes NOX-es from other cellular ROS sources that generate ROS as secondary byproducts along with their main enzymatic function. Controlled ROS generation allows for the implication of NOX-es in the regulation of diverse physiological processes. However, the loss of control over this coordinated ROS production is linked to the onset of various pathological alterations, including liver metabolic disorders [101,102]. Liver-constituted cells express different NOX isoforms. Of note is that each cell type may express different NOX isoforms that execute specialized functions. In hepatocytes, HSCs and endothelial cells, the presence of NOX1, NOX2 and NOX4 was reported, while in Kupffer cells NOX2 was detected. In addition, in hepatocytes, DUOX1 and DUOX2, and in endothelial cells, DUOX2 and NOX5 were described [102,103]. In the hepatic cell line HepG2 cells, the presence of NOX3 was also reported; however, its expression in hepatocytes is yet undetermined [104]. Several studies have addressed the role of NOX enzymes in liver function, but the significance of DUOX-es remains uncovered. The current knowledge concerning the roles of NOX isoforms in liver physiopathology and in NAFLD in particular, is summarized in a later chapter of this review.

## 3. NAFLD, Insulin Resistance and T2DM: Intertwined Pathologies

NAFLD is a multifactorial pathology related to aberrant hepatocyte lipid homeostasis or mitochondrial function, as well as alterations in innate and adaptive immunity and intestinal microbiota composition. The onset of NAFLD is also defined by genetic, nutritional and lifestyle factors. However, two of the major determinants of NAFLD development are insulin resistance (IR) and the presence of T2DM [105]. Indeed, the global prevalence of NAFLD in people suffering from T2DM was reported to be approximately 56% by a recent large-scale meta-analysis [106]. Insulin resistance and T2DM are associated with NAFLD genesis as well as the severity progression (NASH, fibrosis, cirrhosis and hepatocellular carcinoma). The interaction between insulin resistance, T2DM and NAFLD is a bidirectional cause-effect process, and these two conditions act synergically to increase the risk of hepatic and extra-hepatic adverse outcomes, namely sarcopenia, lower cardiac contractility, endothelial dysfunction and chronic kidney injury [6,95,96,107,108,109,110,111]. Employing magnetic resonance spectroscopy, Portillo-Sanchez et al. demonstrated that T2DM is associated with higher hepatic TG content, independent of body mass index, and in the presence of normal transaminase levels [112]. In a cross-sectional analysis of 99.969 apparently healthy, non-diabetic Korean patients, there was an increased risk of NAFLD, determined by ultrasound, with increasing levels of hemoglobin A1c (HbA1c) and insulin resistance, independent of obesity [113]. T2DM predisposes not only to genesis but also to the progression of NAFLD. Younossi et al. showed that an estimated 37% of patients having T2DM and 71% of patients having both T2DM and a biopsied NAFLD developed NASH [106]. The same meta-analysis demonstrated that the estimated advanced fibrosis prevalence among patients with T2DM was 4.8%, and with T2DM and biopsied NAFLD, it was 17% [106]. Transient elastography evaluating liver stiffness indicated a high (up to 17.7%) prevalence of advanced fibrosis in T2DM patients [113,114].

Conversely, several studies have confirmed that NAFLD increases T2DM prevalence. The National Health and Nutritional Examination Survey in the USA showed that among the 5869 screened subjects, T2DM was present in 21% of those with NAFLD (by ultrasound) versus 4.6% in those without [115]. Sung et al., after a 5-year follow-up, observed a doubling of the risk of contracting diabetes when patients were diagnosed with NAFLD after adjustments for other dysmetabolic risk factors [116]. In a large South Korean cohort, Kim et al. found that after a 5 year-follow up, NAFLD was independently associated with the incidence of T2DM [117]. Similar results were brought by Bae et al.: NAFLD was significantly associated with the diagnosis of T2DM, and this association was much stronger among patients with pre-existing impaired fasting glucose (IFG) [118]. Interestingly, it was noticed that the risk of T2DM seems to further increase with the increased ultrasound-based severity of NAFLD, particularly the severity of fibrosis stages. For instance, a study by Bjorkstrom et al. reported a significantly higher risk of T2DM in patients with stage ≥ 3 fibrosis compared to patients with lower fibrosis stages over a mean follow-up period of 18.4 years [119]. The link between NAFLD and T2DM was further affirmed by studies indicating a decreased risk of T2DM incidence over time following an improvement in NAFLD on ultrasound [116,120,121]. 

The biochemical basis of NAFLD development is an imbalance in liver lipid handling, that is, when the rate of hepatic TG synthesis exceeds the rate of hepatic TG catabolism. Enhanced TG synthesis is substantiated by higher hepatic FFA intake and esterification into TGs, as well as by de novo TG synthesis derived from intermediates of carbohydrate and protein metabolism. Triglyceride catabolism includes mitochondrial FFAs β-oxidation as well as export as VLDL [96,122]. The liver receives FFAs from different sources: 60% of hepatic FFAs are derived from the circulation provided by lipolysis of TGs in the WAT, 15% from dietary Apo-E chylomicrons assembled in enterocytes following fat digestion and 25% from de novo lipogenesis (DNL) derived from dietary carbohydrates [123]. To combat hepatocyte FFA overload, FFAs are transported into the mitochondrial matrix via Carnitine-O-Palmitoyl transferase 1 (CPT1), an insulin-inhibited enzyme and undergo β-oxidation. During this process, excessive acetyl-coenzyme A (acetyl-CoA) is addressed to the tricarboxylic acid (TCA) cycle and NADH into the ETC [94]. The critical defensive role of mitochochondrial β-oxidation is supported by several preclinical studies that used genetic and pharmacological approaches to modulate CPT1 activity or the activity of the methylation-controlled J protein (MCJ), an inhibitor of Complex I of the ETC, to attenuate NAFLD [124,125]. The clinical relevance of these findings is supported by data demonstrating that in patients with NAFLD, the levels of CPT1 are decreased, while those of MCJ are increased [125,126]. Similarly, in NAFLD and insulin-resistant states, hepatic FFA export through VLDL is decreased [94,127]. In particular, Dongiovanni et al. showed that the E167K missense mutation in transmembrane 6 superfamily member 2 (TM6SF2) decreased VLDL exports [128]. In this study, 13% of patients who underwent liver biopsy for suspected NASH were carriers of this mutation and were more likely to present with NASH (odds ratio (OR) 1.84) and advanced liver fibrosis (OR 2.08), suggesting that reduced ability to export VLDLs is deleterious for the liver.

The common metabolic element coupling NAFLD and insulin resistance is elevated levels of hepatocyte FFAs. Controversially, however, hepatocyte FFAs are further increased by DNL driven by hyperinsulinemia-related activation of SREBP-1c. Moreover, despite high insulin levels, glucose production and glycogen degradation are high, leading to hyperglycemia, which furthers DNL via two mechanisms. First, hyperglycemia increases acetyl-CoA synthesis, which represents an important substrate for DNL; second, it induces the expression of two central transcription factors, the carbohydrate response element binding-protein (ChREBP) and liver receptor-α (LXRα). ChREBP and LXRα promote the expression of some of the key lipogenic genes, e.g., *Scd1* and *Fasn*, resulting in increased liver fat accumulation [4,6,8]. The clinical relevance of DNL in the development of NAFLD has been demonstrated by studies revealing greater liver fat content in patients with a (rs738409(G)) polymorphism in the Patatin-like phospholipase domain-containing protein 3 (PNPLA3). PNPLA3 plays a role in lipid droplet formation and DNL [129].

Hepatocyte FFA overload activates several intracellular pathways that contribute to the exacerbation of liver insulin resistance. Indeed, accumulated FFAs can aggravate insulin resistance via lysosomal reactions and induction of inflammatory mediators, e.g., the NF-κB and TNFα pathways. FFAs also activate the NOD-, LRR- and pyrin domain-containing protein 3 (NLRP3) inflammasome, leading to the induction of the Caspase-1, and the Interleukin-1β and 18 (IL-1β and IL-18) pathways, thus modulating apoptosis and inflammatory signaling [94]. Furthermore, FFA, transformed into diacylglycerol molecules, can substantiate insulin resistance by activating the signaling cascades of the serine kinases Protein Kinase C-ε (PKCε) and c-Jun-N-terminal kinase (JNK), which hamper insulin receptor activation and subsequent signal transmission by insulin receptor substrates 1 and 2 (IRS1, IRS2) [95,130,131]. Individuals with T2DM showed elevated liver PKC activation and histological analysis of liver biopsies patients with NAFLD demonstrated elevated phosphorylation (activation) of c-jun, the substrate of JNK [130,132].

In addition to FFAs, other lipids, e.g., ceramides, can also contribute to the worsening of IR. A hypercaloric diet can lead to intestinal dysbiosis, which is associated with increased intestinal permeability, permitting the translocation of luminal bacterial lipopolysaccharides (LPS) into the liver. LPS activates Toll-Like Receptor 4 (TLR4), inducing inflammation and ceramide biosynthesis. Ceramides are pro-inflammatory and pro-oxidant molecules that further deteriorate insulin sensitivity. In fact, invalidation of the key enzyme of ceramide synthesis (dihydroceramide desaturase 1 (DES1)) alleviated adiposity and liver steatosis in rodent models [133]. Inhibitors of diverse other elements of the ceramide synthetic pathways are under investigation as promising future pharmaceutical agents in the treatment of NAFLD [95,96,134,135].

In addition to lipids, bile acids are also critical components in the development of liver insulin resistance [136]. Bile acids interact with various nuclear receptors in the intestine, such as the Farnesoid X Receptor (FXR) and the G-protein-coupled bile acid receptor 1 (GPBAR-1). FXR is the master regulator of hepatocyte TG and glucose homeostasis, while GPBAR-1 modulates hepatocyte function by increasing the secretion of Glucagon-Like Peptide 1 (GLP-1) by intestinal L cells and thus instigates insulin secretion from pancreatic islets. Bile acids can also modulate gut microbial composition via the activation of innate immune genes in the small intestine and modify crosstalk events between the liver, microbiota and brown adipose tissue [6,137,138,139]. Bile acid toxicity in hepatocytes is related to oxidative stress [140,141]. Bile acid dysbiosis and the deregulation of their signaling pathways are major contributors to the onset and progression of NAFLD but are beyond the scope of this review [136,142,143]. 

Non-parenchymal hepatic cells (macrophages, lymphocytes, hepatic stellate cells (HSC) and liver sinusoidal endothelial cells (LSEC)) represent approximately 20% of the liver mass. Non-parenchymal hepatic cells are also affected by toxic lipids and contribute to the onset of NAFLD and its progression toward more severe stages.

One of the most important non-parenchymal hepatic cell types is macrophages. The liver contains two major pools of macrophages: embryo-derived resident cells, also known as Kupffer cells (KCs) and monocyte-derived macrophages, recruited from the sinusoidal circulation [144]. Macrophage turnover and function are modulated during the different stages of NAFLD [145,146]. Indeed, the early phase of steatosis development is characterized by a diminution in the number of resident macrophages, leading to increased recruitment and liver accumulation of monocyte-derived macrophages. An association between monocyte recruitment, enhanced liver inflammatory state and more severe stages of NAFLD and NASH is supported by several studies [147,148]. Macrophage inflammatory cytokine secretion is modulated by lipid metabolism. For example, KCs exposed to palmitate in vitro increased the expressions of proinflammatory cytokines (TNFα and IL-6). In contrast, incubation in the presence of polyunsaturated fatty acids (PUFAs) elicits the secretion of the anti-inflammatory cytokine IL-10 [149]. A more complex picture emerged from a study conducted in mice subjected to long-term HFD feeding, with or without cholesterol supplementation. Transcriptomic analysis of liver macrophages isolated from these mice revealed different macrophage populations with different gene regulatory landscapes that included modulation of both pro-and anti-inflammatory cytokine signaling pathways [150]. One of the relevant factors triggering macrophage activation and pro-inflammatory cytokine release is oxidized low-density lipoprotein (oxLDL), which acts as a TLR agonist [151,152]. Interestingly, in vitro experiments suggest that NOX4-derived ROS production is required for oxLDL-induced macrophage death, implying a role for NOX4 as one of the connecting elements between toxic lipids, macrophage function and NAFLD [153].

In addition to macrophages, lymphocytes are also critical participants in the initiation, maintenance and progression of NAFLD [154,155]. In particular, an imbalance in the Th17/Treg lymphocyte populations was noted in a study that analyzed liver biopsies and peripheral blood samples of 31 NAFLD and 30 NASH patients and compared them to 43 healthy controls [156]. The Th17/Treg imbalance is accompanied by augmented signaling of IL-17, a Th17-derived cytokine, and an increase in TNFα production, triggering heightened liver inflammation [157]. In preclinical studies, diet-induced liver pathologies IL-17 promote the onset of NAFLD in mice [158,159]. In addition, elevated IL-17 levels were detected in obese patients with NAFLD [159]. Data derived from mice indicate that liver-infiltrating lymphocytes primarily migrate from mesenteric lymph nodes and thus can serve as links to microbiota-derived toxic lipid signals [160,161]. Redox cues are critical modulators of lymphocyte metabolism, differentiation and function [162,163,164]. In particular, the differentiation of Th17 and Treg cells has been linked to cellular ROS levels, although until now studies aiming to uncover the exact mechanism of action have yielded diverging results [164]. Secretion of IL-17 in vitro from differentiated mouse Th17 cells was inhibited by pro-oxidant treatment [165]. A recent publication identified a distinct subset of inflammatory hepatic Th17 (ihTh17) cells that were sufficient to exacerbate NAFLD development in mice. The accrual of these ihTh17 cells was dependent on PKM2-mediated glycolytic metabolic rewiring [166]. PKM2 modulates ROS production and conversely, ROS can regulate PKM2 activity in diverse cell types [167,168,169,170,171]. The potential role of ROS in the differentiation of ihTh17 cells was not investigated by Moreno-Fernandez et al. but might be an interesting topic for future studies.

HSCs are a pericyte-like cell population of the liver that is localized in the space of Disse and, when activated, plays an essential role in liver fibrosis. In vitro experiments employing human and rat HSC cell lines demonstrated that palmitate incubation promotes HSC activation, inflammation and autophagy [172,173]. Activated HSC also contribute, along with hepatocytes, to the secretion of inflammatory chemokines (CCL5 and CCL20) that are elevated in serum samples of individuals with NAFLD or NASH [174,175]. The relationship between oxidative stress and HSCs is complex and paradoxical. Indeed, ROS play an important role in the proliferation and fibrogenic activation of HSCs. However, ROS also promotes the apoptosis of activated HSCs. This latter effect might reflect a mechanism aimed at mitigating liver injury [176].

LSECs are the most abundant non-parenchymal hepatic cell type. They are different from vascular endothelial cells, as they lack a basement membrane and have several fenestrae that allow the transport of macromolecules. LSECs keep HSCs in a quiescent state [177]. Upon exposure to toxic lipids, LSECs become dysfunctional and undergo a process termed capillarization, characterized by structural changes, such as the development of basal membrane and the loss of fenestrae [178]. LSEC dysfunction seems to precede the onset of hepatic inflammation and fibrosis, underlying the importance of cellular communication between LSECs and other hepatic cells [179]. Indeed, LSEC produce ROS in response to saturated fatty acids that activate the TLR4-NOX1 axis instigating superoxide production [180]. Superoxide can enter circulation and promote HSC activation, proliferation and fibrosis. Conversely, activated HSCs promote LSEC capillarization and further ROS production, thus creating a feedback loop with extended activation of HSCs [181]. The potential role of LSECs’ ROS production in NAFLD/NASH is indicated by a study that reported elevated levels of serum reactive oxygen metabolites in patients with liver pathologies [182].

The protagonist of liver lipid handling imbalance is insulin resistance manifest in diverse organs, most importantly in the WAT and skeletal muscle. The metabolic and hormonal cross-talk between the WAT, the skeletal muscle and the liver are important components in NAFLD development and progression.

White adipose tissue insulin resistance elicits disturbed metabolic crosstalk that promotes enhanced hepatic gluconeogenesis and lipid storage and therefore favors the development of NAFLD [6,94,95,96,107,108,109,111]. Indeed, insulin resistance in WAT generates higher lipolysis and an increased FA shunt to the liver [5,123,183]. WAT insulin resistance is sustained by the continually elevated import of nutrient-derived FFAs into adipocytes. FFA overload generates excessive mitochondrial ETC activity and triggers the onset of oxidative stress, which leads to the activation of inflammatory cytokine secretion and macrophage infiltration into the WAT. Together, these elements perpetuate chronic inflammation and WAT insulin resistance, with deleterious consequences for liver lipid homeostasis. WAT also secretes diverse hormones termed adipokines, which modulate liver metabolism and inflammatory processes. One of the adipokines, leptin, regulates food intake and energy homeostasis and increases insulin resistance and liver fat content. Rotundo et al. showed an association between leptin serum levels and the severity of NAFLD among a population of 1610 patients diagnosed with liver steatosis [184]. Another adipokine, adiponectin, inhibits lipogenesis by downregulating SREBP1c and promoting glucose utilization and FA oxidation through AMP-activated protein kinase (AMPK) signaling. Adiponectin also exerts anti-inflammatory effects and alleviates HFD-induced hepatic inflammation by suppressing MCP-1 expression and macrophage infiltration in mice [185,186]. Matsunami et al. examined the role of adiponectin receptor 2 (AdipoR2) in rat models of NASH [187]. In rats fed with HFD, they found higher rates of fatty liver, inflammation and fibrosis, characteristic of NASH. AdipoR2 expression was significantly decreased, whereas the expression of NOX4 and genes related to the classical NADPH oxidase complex (NOX2, p22^phox^, p47^phox^) were increased. These findings suggest that augmented oxidative stress and inflammation by down-regulation of AdipoR2 may contribute to the progression of NASH and underline the significance of WAT-to-liver crosstalk events in the development and progression of NAFLD [187]. In support of a clinical relevance for adiponectin, several studies have demonstrated low serum adiponectin levels in NAFLD and even lower levels in NASH [188,189].

Skeletal muscle-secreted hormones, myokines, have also been linked to insulin resistance and NAFLD and have been proposed as potential therapeutic targets [190]. Among these, myostatin (MSTN) and irisin are the most studied. Myostatin loss-of-function mutation attenuated liver steatosis in HFD-fed mice [191]. Myostatin contributes to hepatic lipid metabolism disorder by diminishing FA β-oxidation through attenuated PPARα and AMPK signaling [192]. In addition, MSTN promotes liver inflammatory processes by upregulating CD36 and TNFα [191]. From a clinical perspective, circulating MSTN levels are elevated in individuals with obesity, and weight loss reduces MSTN levels and improves insulin sensitivity [193,194]. Myostatin can activate ROS production by a yet undetermined NOX enzyme isoform in muscle cells, although similar activity in hepatocytes has not yet been reported [195]. Irisin exerts a positive effect on hepatocyte lipid metabolism by inhibiting the lipogenic regulators LXRα and SREBP1c and suppressing inflammatory cytokine production through NF-κB [196]. In addition, irisin protects hepatocytes against the onset of oxidative stress in vitro [197]. Controversially, circulating irisin levels are high in individuals with high body mass index (BMI) and insulin resistance, but lower in patients with obesity-related NAFLD [198,199]. These results suggest that elevating irisin production might be a compensatory mechanism that protects the liver against the onset of hepatocyte metabolic and oxidative stress and the development of NAFLD.

The liver communicates with other organs through the release of hepatokines, which are hormone-like proteins primarily secreted by hepatocytes. In healthy conditions, hepatokines relay essential information about the metabolic status of the liver. Hepatokines regulate several biological processes in multiple extrahepatic tissues and mediate physiological benefits in certain circumstances, such as exercise and fasting/refeeding transition [200,201]. Under metabolic duress, hepatocytes produce a dysregulated profile of hepatokines that propagate the effects of NAFLD on whole-body homeostasis. The links between hepatokines, NAFLD and insulin resistance have been summarized in several recent reviews [202,203,204,205]. Most hepatokines play a negative metabolic role and their levels are elevated in NAFLD. Indeed, one of the hepatokines, fibroblast growth factor 21 (FGF21), is considered a biomarker of obesity, T2DM and NAFLD [206]. Fetuin A stimulates pro-inflammatory cytokine production from adipocytes and macrophages and increases insulin resistance. Hepassocin causes insulin resistance and increases hepatic steatosis. Its levels are higher in humans with prediabetes, T2DM and NAFLD [207]. Leukocyte cell-derived chemotaxin 2 (LCT2) impairs insulin signaling and induces pro-inflammatory cytokine expression. Retinol-Binding Protein 4 (RBP4) activates pro-inflammatory pathways and increases insulin resistance. Serum RBP4 levels are higher in individuals with NAFLD and decrease with the regression of liver fat accumulation [208,209]. Finally, Selenoprotein P impairs insulin signaling and glucose homeostasis and is considered a biomarker for T2DM, obesity and NAFLD [210,211]. In contrast to the above-mentioned factors, in NAFLD, the secretion of a few hepatokines is actually decreased, e.g., the Sex-Hormone Binding Protein (SHBG), the Angiopoietin-Like Protein 4 (ANGPTL4) and the adropin. SHBG is inversely associated with liver steatosis and insulin resistance. ANGPLT4 reduces adiposity, increases lipid plasma levels and enhances liver steatosis. Adropin improves insulin sensitivity, hepatic steatosis, whole-body adiposity and insulin resistance.

Of particular interest for this review are some studies that established direct links between hepatokines and redox metabolism. Indeed, RBP4 induces endothelial cell inflammation by inducing the activity of NADPH oxidase and NFκB [212]. A recent study also established that in macrophages, RBP4 primes the NLRP3 inflammasome and promotes inflammatory cytokine (IL-1β, IL-6, TNFα and MCP-1) secretion [213]. The potential connection between RBP4 and NOX-mediated inflammatory signals in macrophages is unknown. Concerning the liver, mice with transgenic overexpression of RBP4 displayed enhanced liver lipid accumulation, which was further aggravated by HFD feeding. The acceleration of steatosis in RBP4 transgenic mice was mainly attributed to reduced mitochondrial content and impaired mitochondrial fatty acid β-oxidation [214]. Selenoprotein P functions as a redox protein through its intrinsic thioredoxin domain and by distributing selenium to GPX proteins; therefore, one would expect to exert beneficial effects on metabolic health [215]. Interestingly, however, in large-scale interventional studies, selenium supplementation was associated with an increased risk of T2DM [216,217]. Similarly elevated serum Selenoprotein P levels were associated with insulin resistance, liver fat deposition and fibrosis [210]. The harmful effects of selenium and Selenoprotein P could be explained by the establishment of reductive stress caused by excess ROS removal, hampering physiological ROS-mediated signaling transmission for the insulin receptor [218].

Together, these data underline the complexity of hepatic and extra-hepatic factors that modulate the relationship between NAFLD, redox imbalance and insulin resistance/T2DM. 

## 4. Oxidative Stress in NAFLD

Oxidative stress plays a crucial role both in the development of hepatocellular injury of NAFLD and in the transitioning from steatosis to NASH, fibrosis, cirrhosis and the development of HCC [219,220]. The onset of oxidative stress is intimately linked to lipotoxicity, defined as the dysregulation of intracellular lipid composition and the extracellular environment [5,123,221]. The accumulation of toxic lipids (e.g., saturated FFAs and ceramides) causes cellular damage by modifying intracellular organelles (mitochondria, ER, lysosomes) as well as by altering intracellular signaling pathways [221].

### 4.1. Mitochondria

Mitochondria is a subcellular organelle considered the powerhouse of the cell. Mitochondria are the main source of ROS generation through the ETC. In physiological conditions, FFAs are oxidized inside the mitochondria through β-oxidation and then, in the TCA cycle, where the energy of their chemical bonds is released as electrons, captured by NAD^+^ and FAD. These molecules, in their reduced forms, NADH and FADH2, are responsible for transmitting the high-energy electrons to the ETC, with the final aim of generating ATP [183,222]. In the presence of aggravated substrate influx, electrons may leak from complex I and III of the ETC and react with molecular oxygen, generating excess ROS capable of damaging other cellular organelles, and leading to their dysfunctions until cellular death [183,222]. ROS overload determines mitochondrial permeability transition pores (MPTP) in the inner mitochondrial membrane, thus inciting a vicious cycle through the induction of MPTP formation and sustained ROS generation. These events promote the onset of chronic inflammation, ATP depletion and cellular death via the activation of caspase-mediated apoptotic pathways [183,222]. In addition, the accumulation of lipid peroxidation products, such as trans-4-hydroxi-2-nonenal (HNE), which directly attacks and inhibits ETC components (e.g., cytochrome c oxidase), can trigger ROS formation, as well as the formation of nitric oxide (NO), leading to direct cellular toxicity [222]. The sum-up of these mechanisms ultimately further aggravates NAFLD and contributes to its progression to more severe stages [5,123,183,222]. On the other hand, mitochondria possess antioxidant protective systems represented by SOD2, Prx proteins, GPX and catalase that combat ROS accumulation. An important role in this antioxidant mechanism is played by the Peroxisome Proliferator-Activated Receptor gamma Coactivator-1alpha (PGC-1α). PGC-1α promotes the transcription of *Sod2* and some ETC components via the activation of Nrf2. Through these actions, PGC-1α enhances superoxide elimination and promotes mitochondrial biogenesis, fostering the restoration of redox homeostasis [5,222]. Nrf2 also regulates the biosynthesis of glutathione and improves redox buffer capacity by augmenting the cellular GSH/GSSG ratio. Moreover, Nrf2 controls the expression of antioxidant genes through specific binding sequences in their promoters, called antioxidant response elements (ARE). Among others, Nrf2 regulates the expression of detoxifying enzymes that eliminate H_2_O_2_ and peroxide radicals from the cytosol, mitochondria and ER [87]. In support of the importance of adequately regulated mitochondrial ROS for the maintenance of cellular health, targeted expression of catalase in the mitochondria in mice prevented age-related mitochondrial damage in myocytes, as well as the onset of HFD-induced cardiomyocyte dysfunction and insulin resistance [223,224]. In hepatocytes in vitro, upregulation of mitochondrial catalase expression was essential to maintain redox buffer capacity and conversely, catalase deletion *in vivo* in HFD-fed mice promoted the onset of NAFLD and insulin resistance through mitochondrial dysfunction [35,225]. Mitochondrial dysfunction is also promoted by oxidative damage inflicted directly by mitochondrial ROS on mitochondrial DNA, which codes for the subunits of the ETC. In addition, appropriate redox control is crucial for the assembly of ETC subunits [226]. Deficient ETC functioning would render hepatocytes more susceptible to oxidative damage upon FFA excessive arrival, thus sustaining a vicious circle between hampered FA oxidative capacity and increased ROS production [123,227]. In line with the results of preclinical investigations, studies indicate that mitochondrial redox balance, appropriate FA oxidation and ETC capacity are important components defense mechanisms against NAFLD in humans [228,229].

### 4.2. Endoplasmic Reticulum and Lysosomes

The ER is a sub-cellular organelle responsible for various crucial cellular functions, including calcium storage, protein synthesis and folding, and protein transportation. Proper protein folding and quality control are achieved by chaperone molecules that reside within the ER and have a high affinity for unfolded proteins. The main ER chaperones are the Binding immunoglobulin Protein/ 78 kDa glucose-regulated protein (BiP or GRP78), the 94 kDa glucose-regulated protein (GRP94), P58IPK, calreticulin and the protein disulfide isomerase (PDI). Under physiological conditions, these chaperones maintain ER homeostasis by preventing protein aggregation, assuming appropriate protein confirmation and disulfide bond formation [230]. Incorrectly folded proteins are excluded from the ER and degraded. However, in metabolic pathologies, high cellular FFA influx can hamper chaperone function, leading to the accumulation of misfolded proteins and triggering the onset of ER stress. Similarly, ER stress can be provoked by other lipotoxic molecules, namely lysophosphatidylcholines [231]. In response to ER stress, the so-called Unfolded Protein Response (UPR) is activated [232]. UPR is mainly mediated through three ER proteins: the inositol-requiring protein 1 (IRE1), protein kinase RNA-like endoplasmic reticulum kinase (PERK) and activating transcription factor 6 (ATF6). Once activated, these proteins induce the transcription and translation processes of chaperones and other molecules to alleviate ER stress and restore ER functions. If the stress is too severe, the UPR-mediated salvage processes are insufficient and multiple apoptotic and inflammatory pathways are engaged [233]. ER damage also increases Sarco/endoplasmic reticulum calcium-ATPase activity, leading to elevated intracellular calcium levels. This activity is linked to the opening of mitochondrial permeability transition pores, causing an increase in ROS production and leading to exaggerated hepatocyte injury [234]. Concerning the redox homeostasis of the ER, the most relevant chaperones are BiP, which can be regarded as the major indirect redox sensor and the protein disulfide isomerase (PDI) that catalyzes protein disulfide bond formation [235]. Lipotoxicity-induced ER dysfunction is linked to the development of NAFLD and in particular, redox ER stress is a hallmark feature of fatty liver disease [236,237,238,239,240]. In particular, BiP/GRP78 expression was elevated in NAFLD human samples [241]. Concerning NOX enzymes, UPR activation enhanced NOX4 levels in peripheral vasculature cells and, conversely, in hepatocytes, NOX4-mediated ROS production extended UPR signaling [242,243].

Lysosomes are membrane-enclosed organelles that contain an array of enzymes capable of breaking down non-functional cell organelles and biomolecules. In addition to this function, lysomomes are essential for nutrient sensing and metabolic adaptation [244]. FFA accumulation, seen in NAFLD, evokes pore formation in lysosome membranes, releasing ROS and other apoptosis-inducing factors [245]. The role of lysosomes and in particular the lysosomal acid lipase, in the development of NAFLD-associated lipid and redox homeostasis disturbances is out of scope of the current review but has been summarized elsewhere [246].

### 4.3. Pro-Inflammatory Signaling Pathways and Intracellular Mediators

ROS, when produced in physiological amounts and in a regulated manner, represent signaling molecules that modulate the normal functioning of the immune response [5]. However, excess, unbridled production of ROS in hepatocytes triggers the formation of Oxidative Stress-derived Epitopes (OSE). OSE are antigen adducts that are then released from damaged hepatocytes and can interact with TLRs on the surface of macrophages. Through this mechanism, OSE abet the onset of pathological innate immune response substantiating liver inflammation observed in NAFLD [102]. In addition to the innate immune response, adaptive immunity also appears to play a role in NAFLD oxidative stress via regulatory Treg lymphocytes. These cells, whose function is to modulate autoimmunity and limit chronic inflammation, are seen to take apoptotic pathways during NAFLD [102]. In addition, excessive FFA arrival in hepatocytes triggers the secretion of pro-inflammatory cytokines IL-1β, IL-6 and TNFα via the activation of NF-κB [5,221]. As a result, monocytes and KCs are recruited, further increasing inflammation and oxidative stress and perpetuating a vicious circle. This cytotoxic mechanism also activates HSCs, thus incrementing scar tissue deposition and the formation of carcinogenic nodules [5,123,183,247]. Production of IL-1β, IL-6 and TNFα is also elevated by specific lipotoxic products, e.g., ceramides and free cholesterol. The latter activates the NLRP3 inflammasome and induces the release of hepatocyte pro-inflammatory cytokines to promote the initiation of KCs and HSCs [123,248,249]. Another factor that reinforces ROS activity and oxidative stress in NAFLD is iron overload. Iron is linked to enhanced lipid peroxidation and the production of malondialdehyde, which activates HSCs and elevates fibrogenesis. Iron also lessens hepatocyte antioxidant capacities by moderating the balance of GSH/GSSG [219]. Last, but not least, during the inflammatory cascade driven by the hepatic accumulation of FFAs, certain pro-oncogenic pathways are activated, such as the signaling mediated by JNK, Janus Kinase 2 (JAK2) and Signal Transducer and Activator of Transcription 3 (STAT3) [221]. Studies have shown that hyperglycaemia predisposes the generation of Advanced Glycation End-products (AGE). These reactive species contribute, through their inner inflammatory activity, to microvascular complications in diabetes. Dehnad et al. found that their receptor (RAGE), leading to pro-inflammatory responses, was significantly induced in patients presenting NASH and T2DM, whereas the AGE receptor 1 (AGER1), customarily responsible for their blood removal and detoxification, was reduced when compared with patients diagnosed with simple hepatic steatosis and no impaired glucose [250].

## 5. NOX Enzymes, Insulin Resistance and T2DM: The Impact on NAFLD Pathogenesis

Oxidative stress acts as a key coupling factor between excess FFA accumulation and hepatic insulin resistance. One of the components of oxidative stress is unbridled ROS generation by NOX enzymes. As detailed in a previous chapter, liver parenchymal and non-parenchymal cells express a variety of NOX isoforms that form a complex and interactive redox network and perturbations in this system are associated with the onset and progression of NAFLD [14,102]. NOX-es are involved in the pathogenesis of hepatocyte lipid accumulation due to mitochondrial oxidative phosphorylation (OXPHOS) dysfunction in mice fed HFD [251]. NOX-es are also involved in activating HSCs, responsible for liver fibrosis, and this mechanism itself can trigger upregulation of diverse NOX isoforms (NOX1, NOX2 and NOX4), creating a vicious cycle and potentially sustaining the progression toward HCC [248,252,253,254,255,256]. In addition, NOX-es exert direct activator effects on the intracellular pathways responsible for neoplastic development through multiple kinase-mediated pathways [102,257].

The impact of different NOX-es on the development of insulin resistance, T2DM and NAFLD was addressed in many investigations. One of the main study approaches was the application of mice deficient in specific NOX isoforms. Of note, most non-phagocytic NOX isoform knock-out mice lack spontaneous, readily observable phenotypes except for NOX3 and its functional accessory subunits (p22^phox^ and NOXO1) and DUOX2. Mice deficient in NOX3 or DUOX2 function present balance problems and congenital hypothyroidism, respectively [258]. Due to the lack of obvious metabolic and liver function-related phenotypes, studies aimed at uncovering the relevant roles of NOX-es in different pathological settings employ targeted modeling of diseases (e.g., HFD or MCD diet feeding). In addition, cell-specific deletions of NOX-es helped uncover the implications of NOX-es in diverse aspects of these medical conditions. While not all studies were directly aimed at investigating the link between NAFLD and NOX-es, these studies still provided significant insights into the NOX-related direct and indirect mechanisms that affected liver function in insulin-resistant and T2DM conditions. A non-exhaustive list focused exclusively on selected experimental NOX-related liver phenotypes is presented in Table 1.

### 5.1. NOX2 and NOX4

The most investigated NOX isoforms in the liver are NOX2 and NOX4. NOX2 appears to play an important role in the development of steatosis and hepatic insulin resistance and is overexpressed in murine models of NASH [102]. Kim et al., investigating the role of NOX2 *in vivo* in mice, found that whole-body NOX2 deficiency attenuated HFD-induced hepatic steatosis. Indeed, after 6 weeks of HFD administration, NOX2-deficient mice not only presented with lower hepatic TG levels, but also with higher hepatic glycogen content, along with attenuated levels of the lipogenic factors SREBP1c and FAS. Taken together, these data indicate that the loss of NOX2 significantly improves hepatic insulin resistance and steatosis. The harmful effect of NOX2 was attributed to the pathological induction of inflammatory signals in hepatic macrophages [261]. A more nuanced role of NOX2 in macrophages was underlined by a study that compared the metabolic phenotype of WT and NOX2 knock-out mice after 8 and 16 weeks of HFD [262]. After 8 weeks, NOX2-deficient mice displayed increased body weight gain, but improved WAT macrophage inflammatory signature, preserved insulin sensitivity and only marginally elevated liver lipid levels. By contrast, after 16 weeks of HFD feeding, NOX2 knock-out mice presented relative WAT atrophy, insulin resistance and marked hepatosteatosis. These data underlined the importance of the WAT-liver axis as a crucial component linking NOX enzyme-derived ROS production, fat storage capacity and insulin resistance. The relevance of immune cell NOX2 in the regulation of metabolic response to a longer-term hypercaloric diet challenge is supported by another study [263]. Mice with myeloid-specific deletions of NOX2 were preserved from the adverse metabolic effects after 16 weeks of HFD feeding. Indeed, myeloid NOX2 knock-out mice displayed lesser body weight gain, lower ER stress in the WAT, and improved lipid profile and insulin sensitivity compared to control mice. The liver phenotype was not specifically examined; however, serum HDL cholesterol levels were elevated, while LDL cholesterol and TG levels were decreased [263]. NOX2 also increases TGF-β phosphorylation in HSCs due to higher peroxynitrite levels, thereby promotes liver fibrosis [254]. An association between NOX2, insulin resistance and liver fibrosis was also confirmed in human studies. Del Ben et al. reported a correlation between insulin resistance and urinary 8-iso-prostaglandin F2α (PGF2α), a reliable tool for indicating subjects with enhanced rates of lipid peroxidation, and hepatic NOX2 levels. Moreover 8-iso-PGF2α was an independent predictor of cytokeratin-18 (CK-18), a marker of apoptosis reflecting liver disease severity. This last finding suggests a possible role for oxidative stress driven by NOX2 and the progression from simple steatosis to NASH [266].

Whole-body NOX4 knock-out mice were employed to study the role of NOX4 in HFD-induced obesity, metabolic alterations, insulin resistance and liver steatosis [265]. After 12 weeks of HFD, NOX4-deficient mice displayed a complex phenotype with markedly elevated adiposity and insulin resistance accompanied by aggravated hepatic lipid accumulation. HFD-induced steatosis was suggested to be related to decreased FFA oxidation (lesser CPT1 expression) with a concomitant increase in the levels of hepatic TG accumulation. Taken together, the data suggest an essential role for NOX4 in appropriate WAT lipid storage and relative protection against ectopic liver fat accumulation [265]. To investigate the direct function of NOX4 in hepatocytes *in vivo*, Bettaieb et al. created mice with hepatocyte-specific ablation of NOX4 (NOX4hepko mice) [243]. Using this knock-out mouse model, the investigators could demonstrate a deleterious role for hepatocyte NOX4 in a model of high fructose and a choline-deficient diet-induced liver injury and fibrosis. In addition, NOX4hepko mice were less insulin resistant compared to the control mice. At the molecular level, next to improved insulin receptor signaling, the NOX4hepko mice also displayed attenuated phosphorylated JNK levels, suggesting lessened stress cellular stress. The detrimental effect of NOX4 on hepatic function was also substantiated by another set of experiments in which mice were kept on a high fructose and choline-deficient diet with or without the concurrent administration of a compound with NOX1/NOX4 dual inhibitor properties (GKT138731) [243]. Mice with inhibitor administration overall had less diet-induced hepatic fibrosis and presented improved glucose tolerance and insulin sensitivity [243]. Concerning the direct effect of NOX4 on hepatocyte insulin sensitivity, an interesting study reported that siRNA-mediated knock-down of NOX4 in McArdle rat hepatoma cells in vitro resulted in selective insulin resistance with diminished insulin effects on lipid and glucose elimination but with concomitant preservation of the lipogenic and MAPK-mediated pathways [267]. In the *in vivo* studies mentioned above [243,265] such discriminative signal transmission was not reported. This discrepancy might be related to compensatory mechanisms that occur in rodent models, where the knock-out effect exists from the very start versus the relatively acute effects observed in cell cultures. Alternatively, this might be attributed to differences between primary hepatocytes and hepatoma cell lines, which display several disparities in their signaling and metabolic response to insulin stimulation [268]. Both *in vivo* studies [243,265] reported lessened markers of liver fibrosis and suggested a link to attenuated HSC activation (ascertained by elevated α-SMA levels). In support of a deleterious role for NOX4 in NASH, histological samples from NASH patients displayed more robust NOX4 staining compared to control samples [243]. NOX4 was also identified as a critical mediator of macrophage M2-type differentiation both in vitro and *in vivo* models and this NOX4-regulated signaling was associated with protection against experimental lung fibrotic conditions [269,270]. A function linking macrophage NOX4 to lung fibrosis was also suggested by data demonstrating elevated NOX4 expression in lung macrophages in asbestos patients [271]. The role of macrophage NOX4 in liver fibrosis has not been directly assessed by currently available studies. NOX4 has also been linked to several aspects of hepatic oncogenesis, including TGF-β signaling [14,272]. However, the neoplastic activity of this isoform is controversial since some studies have demonstrated even antitumoral properties, suggesting that cellular and extracellular signals are important for the context of NOX4 function [273,274]. Indeed, analyzing the NOX enzyme mRNA expression data of 377 HCC and 27 normal liver control samples derived from the Cancer Genome Atlas (TCGA) data portal, Eun et al. found that high NOX4 (and DUOX1) expression correlated with better overall survival [275].

Several studies have demonstrated concomitant, cell type-specific implication of different NOX isoforms in hepatic pathologies. Experimental liver fibrosis induced by carbon tetrachloride was attenuated in both NOX1 and NOX4-knock-out mice and this effect was reproducible by treatment with a dual NOX1/NOX4 inhibitor [276]. The effect of NOX deletion was related to mitigated HSC activation, as attested by lesser expression of profibrotic genes. In addition, both NOX1 and NOX4 protein levels were enhanced in histological analysis of liver biopsies from cirrhotic livers compared to control liver samples [276]. Dietary interventions that promote insulin resistance can induce pathologically elevated activity of different NOX isoforms in specific liver cells and thus engender hepatic oxidative stress. A diet high in fructose raised the expression of NOX2 and NOX4 in the liver. NOX2, expressed in KC and neutrophils, was responsible for the activation of their phagocytic activity, while NOX4 promoted hepatocyte oxidative injury [102]. Furthermore, Cremonini et al. showed that in HepG2 cells, palmitate administration triggered the concurrent upregulation of NOX3 and NOX4, abetting the onset of oxidative stress. Epicatechin and their metabolites, acting by the downregulation of NOX-es, were able to counterbalance palmitate action on protein and lipid oxidation, the stimulation of kinases and the induction of insulin resistance [277]. Similar results were found by Bettaieb et al., who demonstrated that epicatechin could mitigate high-fructose-associated insulin resistance by diminishing the diet-induced upregulation of NOX2 and NOX4 expression in the liver and WAT [278]. In contrast, HFD, in addition to causing the hepatic accumulation of lipids, was observed to activate NOX2, NOX4 and the NOX2 organizer subunit gp47^phox^ in the WAT but exclusively NOX4 in the liver [243]. With reference to gp47^phox^, another interesting molecule, ellagic acid, was able to significantly reduce gp47^phox^ activation, along with an amending effect on hepatic insulin sensitivity [279].

### 5.2. NOX1 and NOX3

Matsumoto et al. observed a significant upregulation of NOX1 in the livers of mice fed a high-fat and high-cholesterol diet for 8 weeks and detected a similar increase in NOX1 expression in NASH patients as well [180]. The deleterious effect of NOX1 was attributed to the elevated levels of protein nitrotyrosine adducts (a marker of peroxynitrite-induced injury) in liver sinusoidal endothelial cells (LSEC) [180]. Sinusoidal endothelial cells inhibit HSC activation thus, diminish fibrogenesis. In another study, macrophage-specific, but not hepatocyte- or HSC-specific, deletion of NOX1 reduced chemically induced hepatic carcinogenesis in mice [260]. The livers of mice with macrophage-specific NOX1 deletion presented elevated expressions of IL-6 and TNFα. Macrophages exposed to conditioned medium from necrotic hepatocytes presented an induced expression of NOX1, followed by an increase in IL-6 and TNFα production. Cytokine production was absent from NOX1-deficient macrophages [260]. Long et al. demonstrated that disactivating Hepatocyte Nuclear Factor 1β (HNF1β) leads to increased intracellular superoxide levels via elevated expression of NOX1, implying HNF1β as a transcriptional repressor for this NOX isoform. Administration of the antioxidant N-Acetyl Cysteine (NAC) significantly decreased knockdown of NOX1, improving both liver fat accumulation and insulin sensitivity [280]. In a study employing the models of carbon tetrachloride and bile duct ligation-induced liver injury in mice, NOX1 was identified as the NOX isoform related to hepatic fibrosis due to its proliferation-inducing effect in HSCs [259]. Similar results were obtained in another study, which also demonstrated more pronounced NOX1 staining in human NASH samples [276].

A study led by Gao et al. investigated the link between NOX3 and insulin sensitivity in the liver. Their aim was to define the role of NOX3-derived ROS in insulin resistance in diabetic (*db*/*db*) mice and HepG2 hepatocytes treated with palmitate. HepG2 cells are nontumorigenic cells with high proliferation rates and an epithelial-like morphology that perform many differentiated hepatic functions. Livers of *db/db* mice displayed higher FFA and ROS levels and an upregulation of *Nox3* mRNA expression along with increased liver fat content and impaired glycogen levels. In line with these *in vivo* results, palmitate-treated HepG2 cells in vitro showed elevated ROS production and NOX3 expression along with decreased insulin receptor signaling related to NOX3-induced activation of the JNK and p38 MAPK pathways [264]. HepG2 cells are also a suitable model for studying the stimulatory effect of insulin on the expression of vascular endothelial growth factor (VEGF), a major promoter of angiogenesis. Heightened angiogenesis is associated with different stages of NAFLD, both in preclinical and clinical settings [281]. Insulin-induced VEGF expression was limited in HepG2 cells with siRNA-mediated NOX3 knock-down along with diminished ROS production and MAPK phosphorylation [104]. NOX3 is primarily expressed in the inner ear, even though its potential upregulation in other tissues and cell types in metabolic disorders has not been specifically addressed. Consequently, the potential role of NOX3 in human NAFLD and insulin resistance is not known and the clinical significance of aforementioned in vitro cell culture and rodent experiments remains elusive.

### 5.3. NOX5

As mentioned earlier, NOX5 is expressed in humans but absent from mice and rats, greatly hindering investigative efforts to uncover its physiopathological role and clinical relevance. NOX5 was demonstrated to play a significant role in the proliferation and fibrosis of human HSC in vitro [282]. In addition, elevated NOX5 expression levels (along with NOX1 and NOX2) were linked to poor survival rates in HCC patients [275]. To address the mechanism of NOX5 action *in vivo*, several NOX5 knock-in mouse models were created with cell-specific expression patterns (endothelial and smooth muscle cells, podocytes, cardiomyocytes and islet β-cells) [283,284,285,286]. Currently, no publicly available study directly addresses the role of NOX5 in the development of NAFLD or NASH. Mice with endothelial cell-specific NOX5 knock-in accumulated more fat tissue but displayed improved glucose tolerance when fed an HFD; however, no data were provided about liver function or liver lipid accumulation [287]. Similarly, mice with β-cell-specific expression gained more weight when subjected to HFD, but their liver status was not assessed [283]. NOX5 promoted renal inflammation and fibrosis [285], and in this context, it might be of interest in future investigations to assess its potential implication in NASH. In addition, NOX5 is expressed in human monocytes and macrophages and governs the monocyte–dendritic cell differentiation process [288,289]. Inflammatory cells and cytokines relay oxidative stress, insulin resistance and NAFLD [1,3]. In particular, serum IL-18 levels have predictive value in obese children with or without NAFLD [290]. In light of these data, further investigations concerning the role of NOX5 in inflammatory processes in NAFLD and NASH are warranted.

## 6. Therapeutic Options: How to Break the Cytotoxic Process

At present, no pharmaceutical therapy targeting NAFLD has been approved. Indeed, currently, the cornerstone of NAFLD therapy is lifestyle modifications aimed at achieving weight loss. Losing 5–6% of body weight is associated with a decrease in liver fat content and more than 10% with an improvement in steatohepatitis and fibrosis [291]. Body weight loss therapies employ dietary interventions and/or the promotion of physical activity. A long-term hypocaloric diet is indicated not only for its effects on body weight, but also because it raises sirtuin and AMPK levels, leading to an increase in PGC1-α levels and sustaining mitochondrial protection [183]. Studies have shown that a Mediterranean diet, rich in antioxidants and mono- and polyunsaturated fatty acids, is recommended for people diagnosed with NAFLD [292]. Reducing red meat and fructose also seems beneficial, as well as increasing caffeine consumption [183]. Physical activity and exercise interventions can also reduce liver fat content and insulin resistance and improve liver and striate muscle mitochondrial function [293,294]. Bariatric surgery is one of the most efficient treatments to achieve weight loss and improve metabolic homeostasis. The effects of the different bariatric surgeries are drastic and entrain a vast range of alterations in cellular signaling, metabolism and function [295]. One of the positive effects identified in patients after bariatric surgery is the diminution of redox stress markers [296,297]. Bariatric surgery improves liver insulin sensitivity, but its connection to improved redox health remains uncharted [298].

The close links between T2DM and NAFLD have drawn attention to the effects of antidiabetic drugs on NAFLD. In preclinical experimental conditions, several antidiabetic drugs have been shown to exert antioxidant effects along with a positive effect on NAFLD, NASH and liver fibrosis. Indeed, in murine models of NASH and fibrosis, the dipeptidyl-peptidase-4 (DPP4) inhibitors (gliptins) significantly decreased parameters of steatosis and inflammation, which was accompanied by a suppression of hepatic transcript levels reflecting inflammation (IL-1β, TNFα and MCP-1) and fibrosis (asserted by α-SMA immunostaining). Gliptins also reduced the number of liver-infiltrating pro-inflammatory (M1) macrophages and polarized them toward anti-inflammatory (M2) phenotypes. This shift was paralleled by decreased hepatocyte NOX-2 expression and mitochondrial ROS production. Taken together, these data imply that gliptins could act as potent suppressors of endotoxin-triggered oxidative bursts despite the lack of direct antioxidant (scavenger) properties [299]. Long et al. found that silencing of DPP4 in hepatocytes diminished HNF1β knockdown-associated superoxide generation by NOX1. DPP4 appeared to contribute to ER stress, hepatic lipid accumulation and insulin resistance [280]. Another interesting class of oral antidiabetic drugs in terms of oxidative stress is SGLT-2 inhibitors (gliflozins). One of these, canagliflozin, administered for 20 weeks, attenuated the development of hepatic steatosis and fibrosis in Western-diet-fed mice. In addition, Shiba et al. observed that after one year of canagliflozin treatment, the number of hepatic tumors was also significantly reduced. Along with its hepatic effects, canagliflozin suppressed the GSH/GSSG ratio in the WAT and attenuated the ROS-induced upregulation of NOX2 and NOX2-related genes in adipocytes [300]. Liraglutide, a glucagon-like peptide-1 (GLP-1) receptor agonist, diminished HFD-induced fatty liver accompanied by a suppression of RAGE and inflammatory cytokines and this effect was reversed by overexpression of NOX2 [301].

In addition to preclinical investigations, the effects of several antidiabetic drugs on NAFLD were also assessed in clinical trials. In this respect, metformin, despite raising hepatic sensitivity to insulin and reducing steatosis in a cohort of NAFLD individuals [302] reached eventually insufficient results in a large meta-analysis [303]. Thiazolidinediones, acting as PPARγ activators, showed some improvements in liver steatosis and lobular inflammation but exerted only a minor effect on fibrosis [304,305]. In the mitochondria, PPARγ agonists enhance the TCA cycle flux [306]. Other PPAR agonists, e.g., the PPARα and -δ-agonist elafibranor and saroglitazar, have been studied with promising but insufficient results [307,308]. Overall, the data for PPAR agonists is conflicting and considering their potential side effects (weight gain, edema and risk of bone fractures), their use in NAFLD is not indicated [6]. GLP-1 receptor agonists showed interesting results in patients diagnosed with NAFLD and T2DM. Liraglutide was associated with a reduction in liver fat content on MRI analysis from the baseline [309]. Liraglutide and semaglutide therapy showed reductions in NASH inflammation but failed to improve fibrosis [110]. SGLT-2 inhibitors were effective in reducing liver fat content but had less impact on inflammation and fibrosis, both in a murine NASH model (canagliflozin) [300] and in clinical trials (dapagliflozin and empagliflozin) [310,311]. Lipophilic bile acids can decrease hepatic lipogenesis and steatosis through the activation of hepatocyte FXR [123]. In this respect, in a clinical trial, administration of obeticholic acid was associated with reduced steatosis, by means of promoting FFA β-oxidation and glycogen synthesis. In addition, obeticholic acid also improved liver fibrosis [312]. Unfortunately, however, another study highlighted an association between obeticholic acid treatment and an increase in plasma levels of TGs and total and LDL cholesterol, questioning its therapeutic value [313].

The redox imbalance observed in NAFLD and the perturbed functions of the components of pro- and antioxidant systems elicited heightened interest in the application of antioxidants as therapeutic means for NAFLD, NASH and liver fibrosis. Vitamin E, for instance, has proven to be efficient in reducing hepatic steatosis and inflammation [314]. However, its clinical application is hindered by the results of several other trials that failed to prove similar efficacy [315]. Indeed, due to the intricate complexity of the interactions between the constituents of redox homeostasis, the efficacy of general antioxidants in NAFLD is still unconvincing [123]. The failure of these non-specific approaches is somewhat predictable, as ROS are physiologically important signal transmitters in various cellular functions and key regulators of cellular “metabolic fitness” [316,317]. 

The redox-related therapeutic approaches mentioned in the chapter above are summarized in Table 2.

The legitimacy of a targeted ROS-focused therapeutic approach is currently well accepted and there are several trials currently underway to establish the potential therapeutic value of redox-based therapies in diverse pathologies To overcome the limitations that led to the failure of previous attempts one need to carefully consider the complexity of redox chemistry and pathophysiology [318]. Currently, edaravone, a potent, small molecule antioxidant that specifically targets OH^−^ radicals, is accepted as a disease-modifying drug for patients with amyotrophic lateral sclerosis (ALS), although its efficiency has recently been questioned [319,320]. Along the lines of targeted antioxidant therapy, much attention has recently been given to novel agents targeting mitochondrial metabolic activity and function, some of them with promising results [123,321]. Another interesting strategy is mitotherapy, which consists of the injection of exogenous mitochondria from hepatoma cells. This protocol decreased lipid content and improved cellular redox balance in a murine model. Interestingly, when the mitochondria were injected into the mice, serum aminotransferase activity and cholesterol levels decreased in a dose-dependent manner. However, further studies are needed to determine the clinical relevance of these mitochondria-targeted approaches [123,229,322].

Taking into consideration the key roles that NOX enzymes play in diverse pathologies, isoform-selective NOX-targeted therapy options provide an increasingly attractive treatment opportunity [12,323,324,325,326]. The World Health Organisation (WHO) recently recognized the validity of this approach and accepted a novel stem, “naxib”, for NOX inhibitors. Currently, several companies are involved in the development of such molecules [325]. Concerning diabetes-related complications specifically, several NOX inhibitors have been developed with varying NOX isoform specificity that have been tested in both preclinical and clinical studies. One of the most advanced compounds is GKT137831, setanaxib, which was marketed as a molecule with a dual NOX1/NOX4 inhibitory effect. However, the specificity of Setanaxib as a *bona fide* NOX inhibitor was later questioned in a detailed pharmacological screen, and its effects were attributed to an unspecified redox mechanism [327]. Nevertheless, Setanaxib showed a suitable safety prolife (NCT03740217) and was employed in several Phase II clinical trials in patients with diabetic nephropathy (NCT0201024) and primary biliary cholangitis (PBC) (NCT03226067) [328]. In these studies, the primary endpoints (albuminuria and gamma-glutamyl transferase (GGT)) were not achieved, but in the PBC study, several secondary endpoints of hepatic markers (alkaline phosphatase, liver stiffness) were significantly improved and the compound now moved to a second Phase II/III randomized, placebo-controlled, double-blind trial with an adaptive design [329]. Another compound, Ewha-18278 (APX-115), inhibits NOX1, NOX2 and NOX4 in the low micromolar range [330]. It has been shown to protect against kidney injury in *db/db* mice [331]. APX-115 was safe in humans (NCT03694041) and has recently advanced to a Phase II clinical trial to assess its effect on renal pathology of T2DM patients, with a primary outcome of mean change in urine albumin to creatinine ratio (NCT04534439).

These results are encouraging and support the clinical validity of NOX-targeted approaches in complex metabolic pathologies; however, the complexity of the NOX-redox circuit still requires careful interpretation of the pre-clinical and clinical data [323,326]. An improved charting of NOX-related signaling and cellular functions is an imperative requirement for future success in the development of novel therapeutic avenues for redox-related pathologies, including NAFLD, insulin resistance and T2DM.

## 7. Conclusions

At present, no pharmaceutical therapy targeting NAFLD is available. The main limitations are the low efficacy of a single-target treatment for such a complex disease and the difficult adaptability of results derived from short-term clinical trials. In the future, combination therapies, such as using a drug with a metabolic mechanism of action with an anti-inflammatory agent, might play an interesting role not only in enhancing the effects of the single components but also minimizing various side effects [123]. In this context, NOX enzymes are promising novel targets, as they exert both metabolism and inflammation regulatory effects, along with their implication in the cellular redox circuit [325]. Unrevealing the relevance of NOX enzymes in the development/progression of NAFLD and their links to insulin resistance/T2DM might offer a step toward a much needed “network pharmacology” attitude aimed at curing causal mechanisms instead of symptom alleviation [10].

## Figures and Tables

**Table 1:** Liver phenotypes of selected NOX-deficient mouse models.

NOX Isoform	Expression in Liver	Treatment-Model	Liver Phenotype	Ref.
NOX1	Hepatocytes HSC	8 weeks HF-HCD-NOX1KO mice	Fibrosis ↓	[180]
		BDL/CCl_4_-NOX1KO mice	Fibrosis ↓	[259]
		DEN inj. 9 mo-NOX1KO mice	Fewer, smaller tumors	[260]
		NOX1^∇Hep^, NOX1^∇HSC^	Similar to WT mice	
		NOX1^∇Mac^	Fewer, smaller tumors	
NOX2	Hepatocytes HSC Kupffer cells	6 weeks HFD-NOX2KO mice	Liver TG ↓	[261]
		8 and 16 weeks HFD-NOX2KO mice	8w: WAT inflammation, Steatosis ↓ Insulin sensitivity preserved 16w: Lipoathrophy Steatosis, Insulin resistance ↑	[262]
		16 weeks HFD-myeloidNOX2KO mice	Insulin resistance ↓ Serum TG ↓ Serum HDL/LDL cholesterol ↑	[263]
NOX3	Hepatic cell line (HepG2)	0.25 mM Palmitate-siNOX3 HepG2 cells	ROS generation, Insulin resistance ↓	[264]
NOX4	Hepatocytes HSC	12 weeks HFD-NOX4KO mice	WAT expansion, Steatosis, Liver a-SMA, Insulin resistance ↑	[265]
		20 weeks High sucrose diet/cholin-deficient diet-NOX4hepko mice and GKT137831 in WT mice	Liver fibrosis, Insulin resistance ↓	[243]

Ref.: Reference; HSC: hepatic stellate cells; HFD: high-fat diet; BDL/CCl_4_: bile duct ligation/ carbon tetrachloride; HF-HCD: high-fat, high-cholesterol diet; DEN: diethylnitrosamine; NOX4hepko: Hepatocyte-specific NOX4 knock-out mice; NOX1^∇Hep^, NOX1^∇HSC^: hepatocyte and hepatic stellate cell-specific NOX1 knock-out mice; myeloidNOX2KO mice: myeloid cell-specific NOX2 knock-out mice.

**Table 2:** Redox-related therapeutic modalities discussed in this review.

Category	Intervention
Lifestyle intervention	Mediterranean diet
	Reduced red meat and fructose consumption
	Physical activity and exercise
Weight loss therapy	Bariatric surgery
Antidiabetic drugs	DPP4 inhibitors (gliptins)
	SLGT-2 inhibitors (glifozins)
	GLP-1 agonists
Bile acids	Obeticholic acid
Antioxidants	Vitamin E
